# Co-Occurrence of Psoriasis/Psoriatic Arthritis and Antiphospholipid Syndrome: A Series of Nine Patients from a Single Centre and Literature Review

**DOI:** 10.31138/mjr.200325.asy

**Published:** 2025-12-31

**Authors:** Vasileios G. Lainis, Olga Katsouli, Panayiotis G. Vlachoyiannopoulos

**Affiliations:** Department of Pathophysiology, Medical School, National and Kapodistrian University of Athens, Athens, Greece

**Keywords:** antiphospholipid syndrome, psoriatic arthritis, psoriasis, interleukin-17, antiphospholipid antibodies

## Abstract

Antiphospholipid syndrome (APS) is associated with pathogenic antiphospholipid antibodies that lead to repeated arterial and venous thromboses and obstetric complications. Psoriatic Arthritis (PsA) is an inflammatory disease characterised by musculoskeletal inflammation and psoriasis (PsO). Evidence for their co-occurrence is scarce. Nine patients with concurrent PsO/PsA and APS, from the APS cohort of the Department of Pathophysiology of LAIKO Hospital of Athens, were identified. APS was the first manifestation in two patients, while seven had PsO/PsA prior to developing APS. Three patients experienced peripheral arthritis, while four showed axial involvement. Regarding APS events, four patients experienced venous thrombosis, five had arterial thrombosis, and three had simultaneous venous and arterial thrombosis. Six patients consistently tested positive for Lupus Anticoagulant, while anti-cardiolipin IgG/IgM antibodies were our cases’ second most common antiphospholipid antibodies. Further research is warranted to determine whether the IL-23/IL-17 axis is a common denominator in the pathogenesis of both diseases.

## INTRODUCTION

Antiphospholipid syndrome (APS) is a thrombo-inflammatory disease characterized by recurrent arterial and venous thrombotic events and obstetrical complications in the presence of pathogenic antiphospholipid antibodies (aPLs).^1^ Psoriatic Arthritis (PsA) is a chronic inflammatory disease characterised by musculoskeletal inflammation and psoriasis (PsO).^2^ Until today, there are only a few case reports highlighting the co-occur-rence of PsA and APS.

## MATERIALS AND METHODS

We conducted a retrospective review of the APS cohort of the Pathophysiology Department of General Hospital of Athens “LAIKO”, who were followed up at the Outpatient Rheumatology Department of the Clinic between 2014 and 2024.

Adult patients, who fulfil both the 2006 revised Sapporo classification criteria for APS^3^ and the 2006 CASPAR (Classification criteria for Psoriatic Arthritis) criteria,^4^ were included. Clinical and laboratory examinations were performed. We followed the International Society on Thrombosis and Haemostasis (ICTH) guidelines for Lupus Anticoagulant (LA) testing and interpretation.^5^ Titres of anti-cardiolipin (anti-CL) IgG and IgM and An-ti-β2glycoprotein (anti-β2GPI) IgG and IgM were measured by standarised ELISA (Enzyme Linked Immuno Sorbent Assay). Positive tests in two occasions, at least 12 weeks apart, were considered as true positive, excluding patients with single positivity.^3^ We excluded all patients with systemic lupus erythematosus or other systemic autoimmune diseases. Clinical, laboratory, and treatment data were collected retrospectively from the medical records. Especially, we reported the type of thrombotic (arterial or venous) events, the pregnancy complications, the type of PsA, emphasising the occurrence of axial involvement, the type of treatment for PsA/PsO and the HLA-B27 status of the patients.

**Figure 1.**
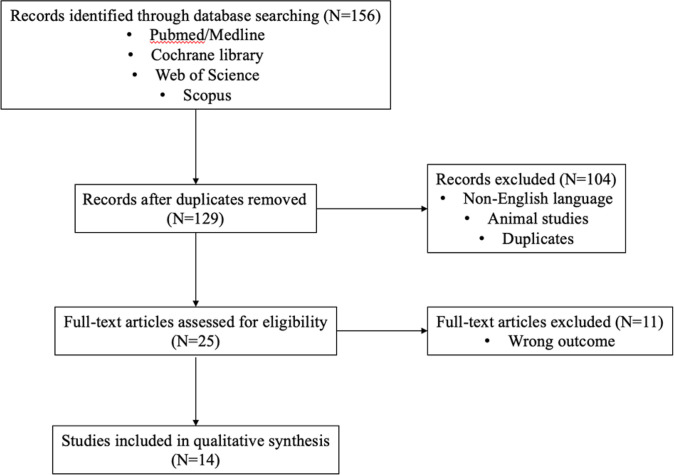
PRISMA-style flow diagram of the search strategy.

Later, a systematic search was conducted in the following databases: PubMed/MEDLINE, Cochrane Library, Web of Science, and Scopus. We used the following keywords: Psoriasis, Psoriatic Arthritis, antiphospholipid syndrome, Hughes syndrome, antiphospholipid antibodies, co-occurrence, co-existence, overlap, and comorbidity. The search was restricted to human studies published in English without date limitations until January 2025. Original articles, case reports, and systematic review articles were included, whereas animal studies, non-English articles, and duplicate publications were excluded^6^ (**Figure 1**). The data were summarised descriptively, focusing on the prevalence, clinical characteristics, and the possible pathophysiological link between PsO/PsA and APS.

## CASES PRESENTATION

In this section, nine cases of patients with concurrent PsO/PsA and APS were presented. **Table 1** summarises their clinical and serological characteristics.

**Table 1.** Clinical and serological characteristics of 9 patients with APS and PsO/PsA.

**Patient**	**Age at APS diagnosis/Gender**	**Baseline disease**	**PsO type**	**PASI baseline at APS diagnosis**	**Type of Arthritis**	**HLA-B27 status**	**First Disease**	**Months until 2nd disease**	**APLs profile**	**APS manifestations**	**Baseline disease treatment**
Pt 1	62/M	PsA	Plaque	8,1	Axial	Positive	PsO	240	Anti-CL IgM/IgG	Ischemic stroke	Anti-IL17
Pt 2	55/M	PsO	Plaque	1	-	Negative	PsO	216	LA Anti-CL IgM/IgG	DVT, PE, MI	Topical
Pt 3	61/M	PsA	Plaque	3,5	Peripheral	Negative	APS	72	LA	Ischemic stroke, PVT	Anti-IL17
Pt 4	52/F	PsA	Plaque	0,9	Peripheral	Negative	PsA	60	LA	DVT	Anti-TNFa Methotrexate
Pt 5	43/F	PsA	Plaque	2,8	Peripheral	Negative	PsO	36	LA Anti-CL IgM	Retinal branch occlusion	Anti-IL17
Pt 6	56/M	PsA	Guttate	0	Axial	Positive	APS	31	Anti-β2GPI IgG	TIA, Amaurosis fugax	Anti-IL17
Pt 7	41/F	PsO	Plaque	3,6	-	Negative	PsO	24	Anti-CL IgM/IgG Anti-β2GPI IgG/IgM	DVT, Amaurosis fugax	Anti-IL17
Pt 8	37/F	PsA	Plaque	1,3	Axial	Positive	PsO	69	LA	Miscarriages (<10 wog)	Anti-IL17
Pt 9	35/F	PsA	Nail	0	Axial	Negative	PsO	96	LA	Miscarriages (<10 wog)	Anti-IL17

M: Male; F: Female; PsO: Psoriasis; APS: Antiphospholipid Syndrome; LA: Lupus Anticoagulant; Anti-CL: Anti-Cardiolipin; Anti-β2GPI: Anti-β2 glycoprotein; DVT: Deep Vein Thrombosis; PVT: Portal Vein Thrombosis; PE: Pulmonary Embolism; TIA: Transient Ischemic Attack; MI: Myocardial Infarction.

### Case 1

A 62-year-old man with a history of PsO (plaque psoriatic lesions on the surfaces of elbows and knees) presented to the emergency department with prosopagnosia and impaired speech. A Brain Computed Tomography (CT) scan revealed an ischemic stroke in the left temporal lobe. Serology testing showed elevated anti-CL IgM: 85 U/ml (<10 U/ml) and anti-CL IgG: 75 U/ml (<10 U/ml). After 12 weeks, testing showed elevated anti-CL-IgG (76 U/ml) and anti-CL-IgM (67 U/ml). The patient was treated with warfarin, targeting an INR of 2.5 to 3. Six months later, the patient reported low back pain, prompting a Magnetic Resonance Imaging (MRI) with sacroiliitis. The HLA-B27 test was positive. He was treated with Secukinumab 300 mg monthly, an anti-IL-17 inhibitor.

### Case 2

A 55-year-old man with a history of plaque PsO presented with swelling in his right thigh. A venous duplex ultrasound revealed deep venous thrombosis (DVT) in the right femoral and popliteal veins. The patient was treated with enoxaparin. Laboratory tests indicated an LA level of 1.76 (normal range <1.3), increased anti-CL-IgM (89 U/ml), and anti-CL-IgG (80 U/ml). After 12 weeks, tests showed an LA level of 1.65, increased anti-CL-IgM (85 U/ml), and anti-CL-IgG (57 U/ml). A diagnosis of APS was established, and warfarin with a target INR of 2.5 to 3 was initiated. Two years later, the patient presented with chest pain. A CT pulmonary angiogram (CTPA) revealed a pulmonary embolism in the left lower lobe segmental branch. The patient was treated with acetylsalicylic acid (ASA) 100 mg and continued warfarin with a new target INR of 3 to 4.

### Case 3

A previously healthy 69-year-old male presented to the emergency department with shortness of breath and chest pain. A CTPA indicated pulmonary embolism in the segmental branches of both lower lobes. Testing showed an LA level of 1.87. A repeat test 12 weeks apart revealed a positive LA of 1.77. The diagnosis of PAPS was established, and warfarin was initiated. Two years later, he suffered from abdominal pain, and a CT scan of the abdomen revealed portal vein thrombosis. He was treated with ASA 100mg daily and continued on warfarin with a new target INR of 3 to 4. One year later, he presented with polyarthritis of the ankles, knees, and wrists, as well as plaque PsO with a Psoriasis Area and Severity Index (PASI) score of 24.4. Treatment with Ixekizumab 80 mg, an anti-IL17 inhibitor, led to a significant clinical improvement.

### Case 4

In 2001, a 57-year-old woman presented with symmetrical arthritis of interphalangeal joints, wrists, and ankles, and plaque PsO. PsA diagnosis was established, and treatment with methotrexate at 15 mg weekly was initiated. One year later, due to partial remission of her arthritis, Etanercept at 50 mg weekly was added to the treatment. In 2012, she presented to the emergency department with pain and swelling in her left calf. Venous duplex ultrasound revealed DVT in the left popliteal vein, and the patient was treated with enoxaparin. Serology testing showed an LA of 1.85. Follow-up tests at 12 weeks indicated an LA of 1.75. APS diagnosis was made, and the patient was treated with warfarin, targeting an INR of 2.5 to 3.

### Case 5

In 2002, a 41-year-old woman with a history of plaque PsO presented with low back pain. An MRI of the sacroiliac joints revealed left sacroiliitis. She was treated with intravenous infliximab at a dose of 5 mg/kg of body weight and methotrexate at 15 mg weekly. Four months later, she experienced visual loss in her left eye. Ophthalmoscopy revealed findings conclusive for branch retinal occlusion. An extensive laboratory evaluation indicated increased anti-CL-IgM levels at 145 U/ml. Repeated tests 12 weeks later showed increased anti-CL-IgM levels at 69 U/ml. APS diagnosis was established, and the patient began treatment with warfarin.

### Case 6

A 52-year-old smoker male with a history of epilepsy, under ASA 100 mg, presented with recurrent episodes of transient vision loss. Imaging tests with CT scan and MRI brain showed no abnormalities. Laboratory evaluation revealed repeated high titres of anti-CL IgG, IgM, and anti-β2GPI IgG, establishing the APS diagnosis. Due to low back pain, an MRI was performed, indicating bilateral sacroiliitis, while the HLA-B27 test was positive. Bimekizumab, an anti-IL17A/F inhibitor, and Warfarin were administered to the patient.

### Case 7

A 40-year-old woman with a history of plaque PsO and episodes of amaurosis fugax under Secukinumab 300mg monthly and ASA 100 mg presented with DVT of the left popliteal vein. In two consecutive measurements, laboratory tests revealed moderate titres of anti-CL IgG, IgM, and anti-β2GPI IgG, IgM. APS diagnosis was made, and warfarin was initiated.

### Cases 8 & 9

Two female patients, aged 34 and 39, with a history of PsA, presented with consecutive pre-foetal deaths. The first patient had plaque psoriasis and sacroiliitis, with the HLA-B27 test positive, while the second experienced nail psoriasis along with sacroiliitis. Both patients were treated with anti-IL-17 inhibitors, effectively managing their disease. Laboratory evaluation for underlying thrombophilia revealed repeated LA tests positive. A diagnosis of obstetric APS was made. Both patients were given pre-pregnancy counselling and treated with ASA 100 mg daily.

## RESULTS

The APS cohort of the Pathophysiology Department of General Hospital of Athens “LAIKO” includes 127 individuals, 70 of whom have primary (PAPS) and 57 secondary (SAPS) APS. Among the SAPS patients, 41 have Systemic Lupus Erythematosus (SLE), while nine suffer from PsO/PsA.

Nine patients (5 females and 4 males), aged between 34 and 59 years (mean age 49.9 years), with concurrent PsO/PsA and APS were investigated. Seven patients suffered from PsA, while the rest presented only with psoriatic lesions without prominent inflammatory arthritis. Most patients (n=7) had non-extensive plaque PsO, with a median baseline PASI at APS diagnosis of 1.3, while one patient had a history of guttate psoriasis and the other nail psoriasis. Two patients had a positive family history of PsO. Three patients experienced peripheral arthritis, while approximately half (n=4) showed axial involvement, confirmed by MRI as sacroiliitis.

In seven patients, the PsO/PsA diagnosis preceded the APS manifestation by a median time of 69 months (min: 24-max: 240). APS was the first manifestation in two patients, 72 and 31 months prior the PsO/PsA appearance.

The majority of patients (n=8) are on biological disease-modifying antirheumatic drugs (DMARDs), including anti-tumour necrosis factor alpha (anti-TNFa) and anti-IL17 for their underlying psoriatic disease, whereas one patient was under topical treatment for PsO. Interestingly, one patient developed APS following treatment with anti-TNFa for PsA.

Regarding the APS clinical events, four patients had experienced venous thrombosis and three had simultaneous venous and arterial thrombosis. Obstetric complications (3 miscarriages <10 weeks of gestation) occurred in two out of the five female patients. Interestingly, none of the patients suffered from both obstetric and thrombotic manifestations.

As for their serological profile, most patients (n=6) consistently tested positive for LA, while, anti-CL IgG/IgM antibodies were the second most common antiphospholipid antibodies in our cases (n=4). None of the patients were triple-aPL positive, three were double-aPL positive and six were single-aPL positive. Seven patients were ANA positive at low titre (≤1:320).

## DISCUSSION

Our previous work has shown that tetramers of CXCL4 chemokine bind two β2GPI molecules, a significant antigen recognised by sera from APS patients. This complex is recognized by aPLs, activating platelets^7^ and endothelial cells.^8^ Furthermore, CD4+ T cells from normal individuals stimulated with supernatants of cultured monocytes treated with anti-β2GPI/β2GPI/CXCL4 mixture released IL-17A.^9^ Moreover, our unpublished preliminary data show that IL-17 has been detected in the plasma and sera of a minority of APS patients. These findings triggered our interest on a potential association between APS, PsO, and/or PsA. As far as we know, this is the largest case series of patients with PsO/PsA and APS. This paper investigates a relatively underexplored area in existing literature, providing important insights into a rare and clinically significant overlap. Since 1973, McDonald et al.^10^ have reported a higher incidence of thrombotic events in patients with PsO; 11.5% of PsO patients experienced at least one thrombotic event.^10^

Later, Fort et al.^11^ found a higher occurrence of anti-cardiolipin antibodies in individuals with PsA.^11^ Conversely, Cassano et al.,^12^ examining the prevalence of aPLs in psoriatic patients, indicated no significant increase in aPLs among those with plaque PsO compared to healthy controls.^12^

The literature only includes a limited number of patients with PsO/PsA and APS (**Table 2**). Yudhishdran et al.^13^ described a 67-year-old woman with a history of plaque PsO who presented with chronic portal vein thrombosis with cavernous transformation and a splenorenal shunt. Tests revealed persistent positive anti-β2GPI antibodies.^12^ In 2022, Imazeki et al.^14^ reported a 75-year-old man with a history of plaque PsO complicated by DVT of the right femoral vein, inferior vena cava, and left popliteal vein. Laboratory investigations showed positive LA and anti-β2GPI antibodies.^14^

**Table 2.** Clinical and serological features of APS and PsO cases in the literature.

	**Age at APS diagnosis/Gender**	**Baseline disease**	**PsO type**	**PASI baseline at APS diagnosis**	**First Disease**	**Months until 2nd disease**	**APLs profile**	**APS manifestations**	**Baseline disease treatment**
Yudhishdran et al.^13^	67/F	PsO	Plaque	unknown	PsO	60	Anti-β2GPI	Portal Vein Thrombosis	Topical
Imazeki et al.^14^	76/M	PsO	Plaque	5,6	PsO	120	LA, Anti-CL IgG, Anti-β2GPI	DVT, PE	Topical

The IL-23/IL-17 axis is crucial in autoimmune pathogenesis, as shown by increased levels of these cytokines in various systemic diseases.^15,16^ Moreover, single nucleotide polymorphisms (SNPs) in the IL-17 and IL-23 receptor genes notably heighten the risk of developing several autoimmune diseases, especially APS.^17^ In APS patients, proinflammatory cytokines induced by IL-17, such as tumour necrosis factor (TNF) and IL-6, were elevated.^18^ However, few studies explore the relationship between the IL-23/IL-17 axis and Th17 cytokine levels in APS patients.

Popovic-Kuzmanovic et al.^19^ found that serum IL-17 and Th17-inducing cytokines levels were significantly elevated in PAPS patients compared to healthy subjects. Additionally, a correlation was observed between IL-17 and IL-23 levels. However, no correlation existed between IL-17 levels and APLs; nonetheless, patients experiencing thrombocytopenia and thrombotic events exhibited higher blood concentrations of IL-17.^19^

Later, Yan et al.^20^ noted a higher Th17/Treg ratio in PAPS and SAPS due to decreased peripheral Treg subsets. Furthermore, in PAPS patients, anti-CL and anti-β2GPI titres were positively correlated with the Th17/Treg ratio, implicating these T-cell subsets in the pathogenesis of APS [20].

APLs are known to promote thrombosis and have been linked to atherosclerosis.^21^ In 2019, Benangiano et al.^22^ examined the influence of CD4+ T cells that target β2GPI in the atherosclerotic lesions of patients with APS and SLE. Their study revealed that in patients with SLE and APS, along with SLE patients who tested positive for APLs, Th1, Th17, and Th1/Th17 infiltrations were observed in atherosclerotic lesions following stimulation with β2GPI antigen, indicating a marked role in accelerated atherosclerosis in SLE and APS.^22^

Recently, a retrospective study evaluating the prevalence of APLs in patients receiving biologic treatment for PsO found that anti-TNFa therapy was associated with a heightened prevalence of APLs compared to patients treated with IL-17 or IL-23 inhibitors. Nevertheless, detecting APLs did not correlate with an increased incidence of thrombotic events.^23^ Although it is the first study to investigate the possible association of anti-TNFa therapy with the prevalence of APLs, it is uncertain whether such treatments should be avoided in these patients. Further studies should be conducted to elucidate these antibodies’ pathogenicity. However, our case series has several limitations. Given its retrospective design, gathering data from the patients’ files was difficult, especially in evaluating the distribution of traditional venous thromboembolism and cardiovascular disease risk factors at the time of the event. Moreover, at this time, psoriatic disease activity (PASI, arthritis-enthesitis-dactylitis assessments, CRP) was assessed from another physician; thus, we couldn’t estimate the additional inflammatory burden on the thrombotic events. Furthermore, the baseline aPLs’ profile in patients with PsO/PsA as first manifestation was unknown. Finally, the small number of reported patients limits the ability to draw definite conclusions about this coexistence. Consequently, our findings should be interpreted cautiously.

## CONCLUSION AND FUTURE PERSPECTIVES

The reported cases highlight a potential overlap between PsO/PsA and APS. To our knowledge this is the largest case series of patients with PsO/PsA and APS. Currently, there is limited evidence regarding the co-occurrence of these conditions. Identifying patients at risk for thrombotic events leads to early intervention and improved clinical outcomes. Further research is warranted to determine whether the IL-23/IL-17 axis serves as a common denominator in the pathogenesis of both diseases or if the presence of APLs may also occur as an epiphenomenon.
